# Onset of efficacy with acute long-acting injectable paliperidone palmitate treatment in markedly to severely ill patients with schizophrenia: post hoc analysis of a randomized, double-blind clinical trial

**DOI:** 10.1186/1744-859X-10-12

**Published:** 2011-04-11

**Authors:** Larry Alphs, Cynthia A Bossie, Jennifer K Sliwa, Yi-Wen Ma, Norris Turner

**Affiliations:** 1 Ortho-McNeil Janssen Scientific Affairs, LLC, Titusville, NJ, USA; 2 Johnson & Johnson Pharmaceutical Research and Development, LLC, Titusville, NJ, USA

## Abstract

**Background:**

This post hoc analysis (trial registration: ClinicalTrials.gov NCT00590577) assessed onset of efficacy and tolerability of acute treatment with once-monthly paliperidone palmitate (PP), a long-acting atypical antipsychotic initiated by day 1 and day 8 injections, in a markedly to severely ill schizophrenia population.

**Methods:**

Subjects entering the 13-week, double-blind trial were randomized to PP (39, 156, or 234 mg [25, 100, and 150 mg eq of paliperidone, respectively]) or placebo. This subgroup analysis included those with a baseline Clinical Global Impressions-Severity (CGI-S) score indicating marked to severe illness. PP subjects received a 234-mg day 1 injection (deltoid), followed by their assigned dose on day 8 and monthly thereafter (deltoid or gluteal). Thus, data for PP groups were pooled for days 4 and 8. Measures included Positive and Negative Syndrome Scale (PANSS), CGI-S, Personal and Social Performance (PSP), and adverse events (AEs). Analysis of covariance (ANCOVA) and last-observation-carried-forward (LOCF) methodologies, without multiplicity adjustments, were used to assess changes in continuous measures. Onset of efficacy was defined as the first time point a treatment group showed significant PANSS improvement (assessed days 4, 8, 22, 36, 64, and 92) versus placebo, which was maintained through end point.

**Results:**

A total of 312 subjects met inclusion criterion for this subgroup analysis. After the day 1 injection, mean PANSS total scores improved significantly with PP (all received 234 mg) versus placebo at day 4 (*P *= 0.012) and day 8 (*P *= 0.007). After the day 8 injection, a significant PANSS improvement persisted at all subsequent time points in the 234-mg group versus placebo (*P *< 0.05). PANSS improvements were greater from day 36 through end point in the 156-mg group (*P *< 0.05) and only at end point in the 39-mg group (*P *< 0.05). CGI-S and PSP scores improved significantly in the 234-mg and 156-mg PP groups versus placebo at end point (*P *< 0.05 for both, respectively); improvement in the 39-mg group was not significant. The most common AEs for PP-treated subjects (≥10%, any treatment group) were headache, insomnia, schizophrenia exacerbation, injection site pain, and agitation.

**Conclusions:**

In this markedly to severely ill population, acute treatment with 234 mg PP improved psychotic symptoms compared with placebo by day 4. After subsequent injections, observed improvements are suggestive of a dose-dependent effect. No unexpected tolerability findings were noted.

## Background

Rapid symptom control in patients with schizophrenia represents an immediate treatment goal, particularly for those who are markedly or severely ill, because acute symptoms are associated with emotional distress, disruptions to the patient's life, and the risk of dangerous behaviors [1]. Knowing when to expect a response with a given antipsychotic is an important clinical consideration in managing these patients. Current data suggest that, although it may take several weeks to achieve full therapeutic effect with an antipsychotic, most of the improvement in psychotic symptoms is often seen within 2 weeks, with an onset of action within the first few days [2-4]. It has also been reported that early antipsychotic response may be indicative of subsequent response in patients with schizophrenia [5].

Achieving an adequate response can be hindered by non-adherence to the treatment regimen, a significant problem in managing patients with schizophrenia [6-8]. Non-adherence within the first days of treatment, which has been reported in nearly one-quarter of patients [9], can impede the onset of efficacy. The use of long-acting injectable antipsychotics obviates the need to take daily medication and may help to improve adherence [1,10].

Reports indicate that long-acting injectable antipsychotics differ with respect to their onset of action. Although it is difficult to compare onset data across studies for the various agents because of the vastly different study designs and populations, there are reports that suggest efficacy can occur as early as within the first few days of treatment [11-14].

Paliperidone palmitate is the palmitate ester of paliperidone [15]. This atypical antipsychotic is a NanoCrystal^® ^(http://www.elandrugtechnologies.com/nanocrystal_technology) suspension of paliperidone palmitate in an aqueous formulation, which is given once monthly by injection (deltoid or gluteal) after a recommended initiation regimen of 234 mg on day 1 and 156 mg on day 8 (both administered in the deltoid) [15,16]. This formulation was designed to rapidly attain therapeutic blood levels [15]. Several controlled clinical studies have confirmed that paliperidone palmitate is effective in controlling symptoms [17-19] and delays time to relapse in patients with schizophrenia [20] without the use of oral antipsychotic supplementation.

This post hoc analysis evaluated the onset of efficacy of acute treatment with paliperidone palmitate and dose effect in markedly to severely ill subjects with schizophrenia over 13 weeks of treatment. Tolerability measures also were evaluated.

## Methods

This was a post hoc analysis of a 13-week, randomized, double-blind, placebo-controlled, multicenter study of three paliperidone palmitate fixed doses in subjects with schizophrenia (ClinicalTrials.gov: NCT00590577; study ID: CRO12550). Methods for the overall study have previously been reported in detail [19].

The overall study was conducted in accordance with the ethical principles that have their origin in the Declaration of Helsinki and that are consistent with good clinical practice and applicable regulatory requirements. The original study protocol was reviewed and approved by an independent ethics committee or an institutional review board at each study site, and all subjects provided written informed consent before entering the study.

### Subjects

Subjects who were at least 18 years of age were eligible for study enrollment if they had a diagnosis of schizophrenia per the *Diagnostic and Statistical Manual of Mental Disorders*, 4th edition (*DSM-IV*) [21], established at least 1 year before screening, and if they had a Positive and Negative Syndrome Scale (PANSS) [22] total score of at least 70 at screening and between 60 and 120, inclusive, at baseline [19]. The criterion for inclusion in this subgroup analysis was a Clinical Global Impressions-Severity (CGI-S) [23] score ≥5 at baseline (markedly to severely ill).

### Study medication

In this report, dosing of paliperidone palmitate is expressed as milligrams. Paliperidone palmitate dosing also may be expressed as milligram equivalents (mg eq) of paliperidone. Paliperidone palmitate doses of 39, 78, 117, 156, and 234 mg are equivalent to 25, 50, 75, 100, and 150 mg eq of paliperidone, respectively [15].

### Study design and randomization

There were two study periods: a screening period of up to 7 days for washout of disallowed psychotropic medications and a 13-week double-blind, fixed-dose treatment period. Subjects were randomly assigned (on a 1:1:1:1 basis) to fixed doses of paliperidone palmitate (39, 156, or 234 mg) or placebo.

On day 1 of the study, all subjects received a deltoid injection of paliperidone palmitate 234 mg or matching placebo. Subjects were required to remain on their previous antipsychotic medication until the day before the first injection of paliperidone palmitate or placebo. On day 8 and monthly thereafter on days 36 and 64, subjects received their assigned treatment per the randomization schedule, injected in the deltoid or gluteal muscle at the investigator's discretion. Oral antipsychotic supplementation was not permitted.

### Inpatient hospitalization

Subjects could be voluntarily hospitalized during the screening period at the investigator's discretion. Subjects were required to be hospitalized from the day of the first injection on day 1 until at least after the second injection of the study drug on day 8.

### Study assessments

The primary efficacy measure was the change in the PANSS [22] total score from baseline to each time point (days 4, 8, 22, 36, 64, and 92) and end point. Onset of efficacy was defined as the first time point at which a given treatment group showed significant PANSS improvement over placebo and maintained significant improvement until end point. Other measures were changes in CGI-S [23] and Personal and Social Performance (PSP) [24] scores from baseline to end point. Safety assessments included the recording and monitoring of treatment-emergent adverse events (AEs) and laboratory tests. Additionally, subjects were assessed for movement disorders with the Simpson-Angus Scale (SAS) [25], Barnes Akathisia Rating Scale (BARS) [26], and Abnormal Involuntary Movement Scale (AIMS) [27].

### Analysis set

In this post hoc analysis, all efficacy and safety analyses were performed on the intent-to-treat (ITT) analysis set, which included all randomized subjects who received at least one dose of double-blind study medication and had both baseline and at least one post-baseline efficacy assessment.

### Statistical analysis

Analyses compared the three paliperidone palmitate groups (note that per the study design the paliperidone palmitate groups were pooled for days 4 and 8) with the placebo group at baseline at each time point (including end point), using the ITT analysis set. Mean (SD), median, minimum, and maximum were used for summary of continuous variables; percentage and frequency were used for categorical variables. Between-treatment-group differences in continuous variables were evaluated using an analysis of covariance (ANCOVA) model, with treatment and country as factors and baseline score as a covariate. Changes from baseline are presented as least-squares (LS) means and standard errors (SEs). Change from baseline in PANSS total score was further evaluated for all subjects using a mixed model with time, country, treatment, and treatment-by-time interaction as factors and baseline value as a covariate. An unstructured variance-covariance matrix was employed for this analysis. Between-treatment-group differences in benzodiazepine use and response rates were evaluated using the Cochran-Mantel-Haenszel test, controlling for country. LS mean changes from baseline to end point and their 95% confidence intervals for effect sizes of treatment versus placebo were calculated using Cohen's *d *methodology, and between-group differences were evaluated using the ANCOVA model described above. All statistical tests were two sided, and no adjustments were made for multiplicity. Last-observation-carried-forward (LOCF) methodology was used.

## Results

### Baseline demographic and clinical characteristics

Of the 652 subjects in the original study, 312 (47.9%) had marked to severe baseline illness as defined by the CGI-S scale. Among these subjects, 88.1% had CGI-S ratings of marked illness and 11.9% had severe illness. Baseline demographic and clinical characteristics appeared similar between the three paliperidone palmitate groups and the placebo group (Table 1). With the exception of the PANSS total score and baseline CGI-S score, baseline demographics and clinical characteristics were similar to those of the overall study population. The mean (SD) PANSS total score at baseline was 87.1 (11.2) for the overall population versus 94.7 (8.9) for the markedly to severely ill population.

**Table 1:** Baseline demographic and clinical characteristics

Baseline demographics/patient characteristics	**Placebo**, n = 83	Paliperidone palmitate		
		
		234/39 mg, n = 72	234/156 mg, n = 72	234/234 mg, n = 85
Age in years, mean (SD)	40.3 (11.2)	40.1 (10.2)	38.4 (10.6)	39.0 (11.0)

Sex, n (%)				

Male	56 (67.5)	50 (69.4)	47 (65.3)	60 (70.6)

Female	27 (32.5)	22 (30.6)	25 (34.7)	25 (29.4)

Race, n (%)				

Caucasian	39 (47.0)	40 (55.6)	35 (48.6)	40 (47.1)

African American	36 (43.4)	24 (33.3)	26 (36.1)	35 (41.2)

Asian	8 (9.6)	5 (6.9)	10 (13.9)	6 (7.1)

Other	0 (0)	3 (4.2)	1 (1.4)	4 (4.7)

Age at diagnosis in years, mean (SD)	24.9 (8.1)	24.2 (6.8)	25.8 (8.7)	24.3 (8.0)

Baseline PANSS total score, mean (SD)	92.6 (9.2)	95.8 (8.9)	94.5 (7.9)	96.0 (9.2)

Baseline CGI-S score, n (%)				

Marked (= 5)	73 (88.0)	60 (83.3)	68 (94.4)	74 (87.1)

Severe (≥6)	10 (12.1)	12 (16.7)	4 (5.6)	11 (12.9)

Prior hospitalization for psychosis, n (%)				

None	5 (6.0)	5 (6.9)	6 (8.3)	7 (8.2)

1	19 (22.9)	10 (13.9)	9 (12.5)	11 (12.9)

2	16 (19.3)	13 (18.1)	18 (25.0)	17 (20.0)

3	9 (10.8)	12 (16.7)	13 (18.1)	12 (14.1)

≥4	34 (41.0)	32 (44.4)	26 (36.1)	38 (44.7)

Disposition				

Completed, n (%)	32 (38.6)	35 (48.6)	36 (50.0)	43 (50.6)

Discontinued, n (%)	51 (61.5)	37 (51.4)	36 (50.0)	42 (49.4)

Reasons for discontinuation				

Lack of efficacy	24 (28.9)	16 (22.2)	14 (19.4)	14 (16.5)

Withdrew consent	14 (16.9)	10 (13.9)	11 (15.3)	21 (24.7)

Adverse event	7 (8.4)	6 (8.3)	7 (9.7)	5 (5.9)

Lost to follow-up	4 (4.8)	5 (6.9)	3 (4.2)	1 (1.2)

Other	2 (2.4)	0 (0)	1 (1.4)	1 (1.2)

Each paliperidone palmitate subject received 234 mg of paliperidone palmitate on day 1 and then their assigned dose day 8 and monthly thereafter.

CGI-S = Clinical Global Impression-Severity scale; PANSS = Positive and Negative Syndrome Scale.

### Subject disposition

Completion rates were 38.6% of the placebo group and 48.6%, 50.0%, and 50.6% of the 39-mg, 156-mg, and 234-mg paliperidone palmitate groups, respectively (Table 1). The most common reasons for treatment discontinuation were lack of efficacy in the placebo (28.9%), paliperidone palmitate 39-mg (22.2%), and paliperidone palmitate 156-mg (19.4%) groups and withdrawal of consent in the paliperidone palmitate 234-mg group (24.7%).

### Benzodiazepine use

Proportions of subjects who used benzodiazepines during the study were 63.9% in the placebo group and 69.4%, 66.7%, and 64.7% in the paliperidone palmitate 39-mg, 156-mg, and 234-mg groups, respectively. No significant differences were observed between the treatment groups versus placebo in benzodiazepine use.

### Efficacy

**Figure 1:**
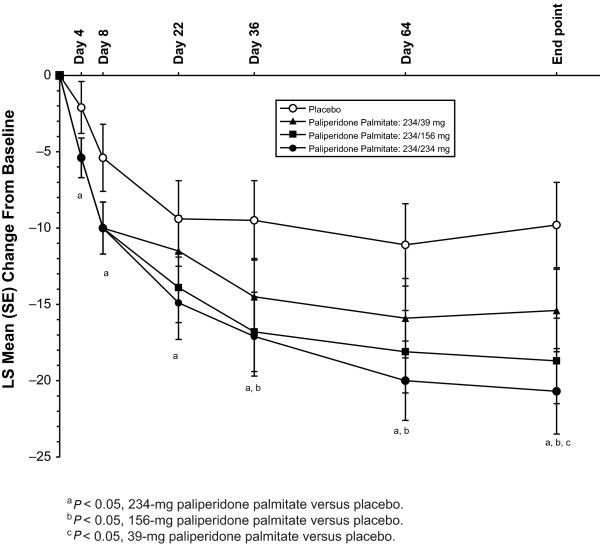
**Least-squares (LS) mean Positive and Negative Syndrome Scale (PANSS) total score change from baseline for the paliperidone palmitate dose groups versus placebo group (last-observation-carried-forward [LOCF] analysis)**. All paliperidone palmitate-treated subjects received paliperidone palmitate 234 mg on day 1 and then received their assigned treatment on days 8, 36, and 64. SE = standard error.

**Figure 2:**
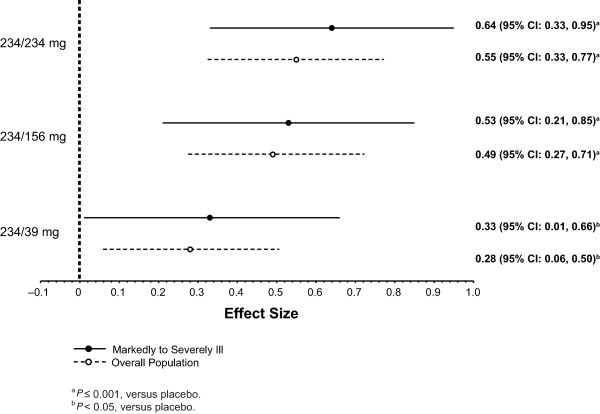
**Effect size for change from baseline to end point on Positive and Negative Syndrome Scale (PANSS) total score for markedly to severely ill subjects and overall study population**. CI = confidence interval.

**Figure 3:**
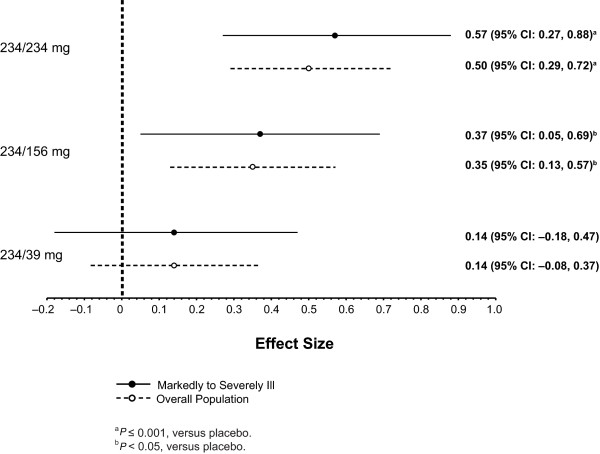
**Effect size for change from baseline to end point on Clinical Global Impressions-Severity (CGI-S) score for markedly to severely ill subjects and overall study population**. CI = confidence interval.

**Figure 4:**
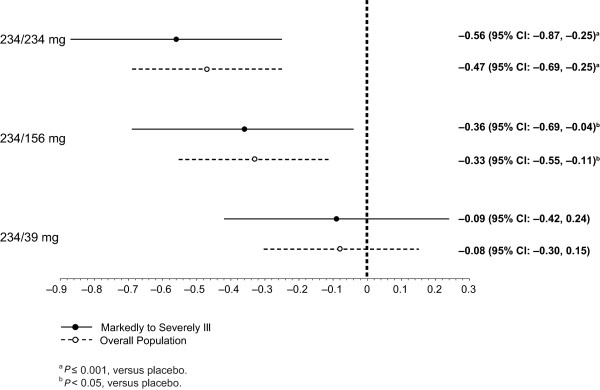
**Effect size for change from baseline to end point on Personal and Social Performance (PSP) score for markedly to severely ill subjects and overall study population**. CI = confidence interval.

After the day 1 injection, LS mean PANSS total scores improved significantly with paliperidone palmitate (all received 234 mg) versus placebo at day 4 (*P *= 0.012) and day 8 (*P *= 0.007) (Figure 1). After the day 8 injection of the assigned dose, a significant PANSS improvement was seen at all subsequent time points in the 234-mg group versus placebo (*P *< 0.05). PANSS improvement versus placebo was greater from day 36 through end point in the 156-mg group (*P *< 0.05) and only at end point in the 39-mg group (*P *< 0.05) (Table 2). LS mean (SE) CGI-S and PSP scores improved significantly by day 36 (-1.4 [0.2] and 14.4 [1.9], respectively) in the 234-mg paliperidone palmitate group (*P *< 0.05) versus placebo. LS mean (SE) CGI-S scores were improved significantly at day 36 and at end point, and LS mean (SE) PSP scores improved significantly at end point for the 156-mg paliperidone palmitate group versus placebo (*P *< 0.05). Improvement in CGI-S and PSP scores in the 39-mg group did not reach significance at any time point. Corresponding effect sizes for paliperidone palmitate versus placebo at end point for the PANSS (Figure 2), CGI-S (Figure 3), and PSP (Figure 4) showed similar results.

**Table 2:** Efficacy assessments from baseline to end point (LOCF analysis)

	**Placebo**, **n = 83**	Paliperidone palmitate		
		
Efficacy measure		234/39 mg, n = 72	234/156 mg, n = 72	234/234 mg, n = 85
PANSS total score				

Baseline, mean (SD)	92.6 (9.2)	95.8 (8.9)	94.5 (7.9)	96.0 (9.2)

LS mean (SE) change from baseline	-9.8 (2.8)	-15.4 (2.7)	-18.7 (2.8)	-20.7 (2.8)

*P *value for LS mean (vs placebo)	--	0.046	0.001	<0.001

CGI-S score				

Baseline, mean (SD)	5.1 (0.3)	5.2 (0.4)	5.1 (0.2)	5.1 (0.3)

LS mean (SE) change from baseline	-0.9 (0.2)	-1.1 (0.2)	-1.3 (0.2)	-1.5 (0.2)

*P *value for LS mean (vs placebo)	--	0.387	0.023	0.003

PSP score				

Baseline, mean (SD)	42.9 (10.1)	41.3 (10.4)	44.7 (11.5)	41.0 (10.1)

LS mean (SE) change from baseline	10.3 (2.2)	11.5 (2.1)	15.1 (2.2)	17.7 (2.2)

*P *value for LS mean (vs placebo)	--	0.597	0.028	0.0005

Each paliperidone palmitate subject received 234 mg of paliperidone palmitate on day 1 and then their assigned dose day 8 and monthly thereafter.

CGI-S = Clinical Global Impression-Severity scale; LOCF = last-observation-carried-forward; LS = least-squares; PANSS = Positive and Negative Syndrome Scale; PSP = Personal and Social Performance.

Results were similar using the mixed-model repeated measures analysis of the PANSS total score (overall LS mean (SE) treatment difference versus placebo: 39-mg treatment group -4.0 (1.9), *P *= 0.034; 156-mg treatment group, -6.5 (1.9), *P *< 0.001; 234-mg treatment group, -6.4 (1.8), *P *< 0.001). The percentage of subjects who achieved a response at end point was 15.7% in the placebo group and 36.1% (*P *= 0.014 versus placebo), 34.7% (*P *= 0.026), and 41.2% (*P *< 0.001) for the 39-mg, 156-mg, and 234-mg paliperidone palmitate groups, respectively.

### Safety

Reported AEs, discontinuations due to AEs, and extrapyramidal symptom (EPS)-related AEs are shown in Table 3. The most common AEs in paliperidone palmitate-treated subjects (≥10% in any treatment group) were headache, insomnia, schizophrenia exacerbation, injection site pain, and agitation.

**Table 3:** Treatment-emergent adverse events (AEs)

Adverse events, n (%)	**Placebo**, **n = 83**	Paliperidone palmitate		
		
		**234/39 mg**, **n = 72**	**234/156 mg**, **n = 72**	234/234 mg, n = 85
Patients with ≥1 AE	57 (68.7)	49 (68.1)	53 (73.6)	55 (64.7)

Discontinuation due to AEs	7 (8.4)	6 (8.3)	7 (9.7)	5 (5.9)

Most common AEs*				

Insomnia	13 (15.7)	8 (11.1)	5 (6.9)	10 (11.8)

Headache	7 (8.4)	11 (15.3)	9 (12.5)	6 (7.1)

Schizophrenia exacerbation	8 (9.6)	6 (8.3)	10 (13.9)	4 (4.7)

Injection site pain	5 (6.0)	10 (13.9)	2 (2.8)	7 (8.2)

Anxiety	6 (7.2)	3 (4.2)	5 (6.9)	7 (8.2)

Agitation	8 (9.6)	8 (11.1)	2 (2.8)	2 (2.4)

Akathisia	5 (6.0)	1 (1.4)	5 (6.9)	5 (5.9)

Psychotic disorder	6 (7.2)	3 (4.2)	4 (5.6)	2 (2.4)

Dizziness	1 (1.2)	1 (1.4)	5 (6.9)	3 (3.5)

Upper respiratory tract infection	1 (1.2)	0	1 (1.4)	5 (5.9)

Constipation	3 (3.6)	2 (2.8)	4 (5.6)	0

Patients with ≥1 EPS-related AE	7 (8.4)	5 (6.9)	9 (12.5)	10 (11.8)

Each paliperidone palmitate subject received 234 mg of paliperidone palmitate on day 1 and then their assigned dose day 8 and monthly thereafter.

*Defined as ≥5% in any one group.

EPS = extrapyramidal symptom.

Mean SAS, BARS, and AIMS scores were low (≤1) in all groups at baseline and end point. No significant differences for paliperidone palmitate groups versus placebo were observed in the mean change from baseline to end point score for these movement disorder rating scales.

The LS mean (SE) weight change (kg) from baseline to end point was 0.4 (0.7) in the placebo group versus 0.7 (0.6) in the paliperidone palmitate 39-mg group (*P *= 0.652), 0.8 (0.7) in the 156-mg group (*P *= 0.513), and 1.1 (0.7) in the 234-mg group (*P *= 0.269). No significant between-group differences were observed for the change from baseline to end point in triglyceride, high-density lipoprotein, or low-density lipoprotein levels for paliperidone palmitate versus placebo. Small but significant differences were observed in LS mean (SE) change from baseline to end point in plasma glucose levels (mmol/l) between placebo (-0.31 [0.17]) and the 39-mg (0.05 [0.16]; *P *= 0.033) and 156-mg (0.02 [0.16]; *P *= 0.049) paliperidone palmitate dose groups; the difference between the placebo group and the 234-mg paliperidone palmitate group was not significant (-0.13 [0.2]; *P *= 0.253).

Prolactin levels increased significantly with paliperidone palmitate versus placebo in males and females in all dose groups (*P *≤0.001, Table 4). In the markedly to severely ill population, one subject in the paliperidone palmitate 39-mg group had an AE potentially related to prolactin (ejaculation disorder).

**Table 4:** Least-squares (LS) mean (SE) change from baseline in prolactin levels (ng/ml) at end point

Variable	Placebo, n = 83	Paliperidone palmitate		
		
		234/39 mg, n = 72	234/156 mg, n = 72	234/234 mg, n = 85
Males				

n	56	50	46	60

Baseline, mean (SD)	31.7 (21.1)	28.7 (21.2)	30.2 (25.2)	28.4 (19.0)

LS mean (SE) change from baseline	-25.0 (4.1)	1.3 (3.9)	0.6 (4.1)	2.6 (3.9)

*P *value for LS mean (vs placebo)	--	<0.001	<0.001	<0.001

Females				

n	27	22	25	25

Baseline, mean (SD)	81.8 (50.9)	76.2 (53.0)	85.7 (56.3)	80.0 (67.8)

LS mean (SE) change from baseline	-45.9 (17.1)	17.8 (16.3)	17.8 (16.8)	42.9 (17.9)

*P *value for LS mean (vs placebo)	--	0.001	<0.001	<0.001

Each paliperidone palmitate subject received 234 mg of paliperidone palmitate on day 1 and then their assigned dose day 8 and monthly thereafter.

## Discussion

This subgroup analysis of patients with moderate to severe schizophrenia showed that acute treatment with long-acting paliperidone palmitate, compared with placebo, improved symptoms as early as day 4 (the first post-baseline time point), with improvement continuing at day 8. Thus, these findings support rapid symptom control after a day 1 deltoid injection of paliperidone palmitate 234 mg in these patients. The demonstrated onset of response within a week of initiating treatment with long-acting paliperidone palmitate is consistent with that documented with a number of other antipsychotics, including oral agents [2,3,11,13,28-30]. Of note, oral antipsychotic supplementation was not permitted in this paliperidone palmitate trial. Thus, improvement cannot be attributed to additive effects with other drugs.

After the injections of paliperidone palmitate 234, 156, or 39 mg at day 8 and monthly thereafter, data at the subsequent time points suggested a dose-dependent response in this more severely ill subpopulation. This conclusion is supported by improvements in symptomatology (PANSS total scores) at each time point, as well as with measures of clinical status (CGI-S) and functioning (PSP) at end point. The 234-mg group showed the most robust effect in this subgroup by all measures (Figures 1, 2, 3, and 4), although the 156-mg group showed substantial and statistically significant improvement by these measures (except for non-significant improvement versus placebo on PANSS total change on day 22). In contrast, the 39-mg group did not show significant improvement compared with placebo except at end point on PANSS total score. Further, the CGI-S and PSP improvements at this lowest dose never reached statistical significance compared with placebo. It is relevant to note here that the paliperidone palmitate initiation regimen used in this study differed somewhat from the recommended regimen of deltoid injections with 234 mg on day 1 and 156 mg on day 8 [16]. Thus, the day 8 injections in the gluteal muscle (versus deltoid), with 39 mg (lower than the recommended day 8 dose of 156 mg), may have resulted in particularly low paliperidone palmitate blood levels [15] and may have contributed to some of the observed results. Nevertheless, it is not unreasonable to find that higher doses may be needed in many moderately to severely ill patients and they should be considered when managing such patients.

The dose-dependent trend in improvements observed in this subgroup was similar to that observed in the overall study population, although it appeared to be consistently more robust in this markedly to severely ill subgroup. This trend was observed for improvements in the PANSS, CGI-S, and PSP scores (Figures 2, 3, and 4), as well as in discontinuation rates for lack of efficacy. This finding may be expected with an efficacious treatment in patients who are particularly ill or symptomatic and thus have more room for improvement (that is, regression to the mean phenomenon). Of note, although the markedly to severely ill subjects in this report were a symptomatic subgroup, on average they were not a resistant one.

Tolerability findings in this subgroup did not suggest that AEs were generally more common with the highest dose of 234 mg. However, data were consistent with a dose-dependent trend for anxiety and upper respiratory tract infection. Also, EPS-related AE rates were the lowest at 39 mg and comparable at 156 mg and 234 mg. In the markedly to severely ill subgroup, there was a trend for a dose-dependent increase in weight, with a mean increase of 1.1 (0.7) kg in the highest dose group at end point (*P *= 0.269 vs placebo). The incidence of EPS-related AEs and changes in weight were similar to those of the overall study population [16]. Consistent with the known pharmacology of paliperidone, mean prolactin levels increased from baseline to end point in the markedly to severely ill population, with a greater increase observed in women. The incidence of AEs potentially related to prolactin in this subpopulation was low (≤1%) and similar to that observed in the overall study population [19] and in other clinical trials of paliperidone palmitate [17,18,31]. Thus, no unexpected tolerability findings were noted in this subgroup analysis overall.

This post hoc analysis was performed with data from a trial that was not specifically designed to study markedly to severely ill subjects. In the original trial, study inclusion criteria for symptomatology required only that subjects have PANSS scores of at least 70 at screening and 60 to 120 at baseline. Nonetheless, 48% of subjects met our subgroup criterion for at least marked illness at entry, providing a sufficient sample for study. However, data were not available to demonstrate how long subjects were at this level of illness severity. Also, most of the subjects in this analysis were rated as markedly ill, and as such, these data may not generalize to those with more severe illness. Of note, within this subgroup of markedly to severely ill subjects, 49% to 62% of each treatment group discontinued before completion of the 13-week study period. The rates in the overall population were 46% to 57%, respectively [19]. Although such premature discontinuation rates are not unexpected in trials of schizophrenia, this must be considered when interpreting results.

## Conclusions

In this post hoc analysis of a 13-week study, acute treatment with paliperidone palmitate initiated at 234 mg, without oral antipsychotic supplementation, improved symptoms (as determined by PANSS score) compared with placebo by day 4 in markedly to severely ill patients with schizophrenia. After the subsequent day 8 and monthly injections of 39, 156, or 234 mg, improvements in symptomatology, clinical status, and functioning exhibited a dose-dependent trend, with the least robust effect at 39 mg, significant improvements at 156 mg, and the most robust effect at 234 mg. No unexpected tolerability findings were noted. These findings suggest that acute treatment with paliperidone palmitate is an effective and tolerated treatment option for markedly to severely ill patients with schizophrenia and provide dosing data that can help guide clinicians when managing these patients.

## Competing interests

LA, CAB, JKS, and NT are employees of Ortho-McNeil Janssen Scientific Affairs, LLC, and Johnson & Johnson stockholders. Y-WM is an employee of Johnson & Johnson Pharmaceutical Research and Development, LLC, and a Johnson & Johnson stockholder.

## Authors' contributions

LA, CAB, JKS, and NT participated in the design of this subanalysis. Y-WM performed the statistical analyses. All authors (LA, CAB, JKS, Y-WM, and NT) were involved in developing the drafts of the manuscript, participated in its subsequent revisions, and read and approved the final manuscript.
